# Oligonol Ameliorates CCl_**4**_-Induced Liver Injury in Rats via the NF-Kappa B and MAPK Signaling Pathways

**DOI:** 10.1155/2016/3935841

**Published:** 2015-12-21

**Authors:** Jeonghyeon Bak, Nam Kyung Je, Hae Young Chung, Takako Yokozawa, Sik Yoon, Jeon-Ok Moon

**Affiliations:** ^1^College of Pharmacy, Pusan National University, Busan 609-735, Republic of Korea; ^2^Graduate School of Science and Engineering for Research, University of Toyama, Toyama 930-0194, Japan; ^3^Department of Anatomy, College of Medicine, Pusan National University, Yangsan 626-870, Republic of Korea

## Abstract

Oxidative stress is thought to be a key risk factor in the development of hepatic diseases. Blocking or retarding the reactions of oxidation and the inflammatory process by antioxidants could be a promising therapeutic intervention for prevention or treatment of liver injuries. Oligonol is a low molecular weight polyphenol containing catechin-type monomers and oligomers derived from lychee fruit. In this study, we investigated the anti-inflammatory effect of oligonol on carbon tetrachloride- (CCl_4_-) induced acute hepatic injury in rats. Oral administration of oligonol (10 or 50 mg/kg) reduced CCl_4_-induced abnormalities in liver histology and serum AST and serum ALT levels. Oligonol treatment attenuated the CCl_4_-induced production of inflammatory mediators, including TNF-*α*, IL-1*β*, cyclooxygenase-2 (COX-2), and inducible nitric oxide synthase (iNOS) mRNA levels. Western blot analysis showed that oligonol suppressed proinflammatory nuclear factor-kappa B (NF-*κ*B) p65 activation, phosphorylation of extracellular signal-regulated kinase (ERK), c-Jun NH_2_-terminal kinase (JNK), and p38 mitogen-activated protein kinases (MAPKs) as well as Akt. Oligonol exhibited strong antioxidative activity *in vitro* and *in vivo*, and hepatoprotective activity against *t*-butyl hydroperoxide-induced HepG2 cells. Taken together, oligonol showed antioxidative and anti-inflammatory effects in CCl_4_-intoxicated rats by inhibiting oxidative stress and NF-*κ*B activation via blockade of the activation of upstream kinases including MAPKs and Akt.

## 1. Introduction

Liver inflammation is a common response to various types of chronic liver injury. In the initial stages of inflammation, hepatocytes, Kupffer cells, platelets, and leukocytes are activated and produce reactive oxygen species (ROS) and inflammatory mediators such as platelet-derived growth factor, transforming growth factor-*β* (TGF-*β*), connective tissue growth factor, and tissue necrosis factor-*α* (TNF-*α*). These factors probably act as paracrine mediators to activate quiescent hepatic stellate cells (HSCs) that are localized in the perisinusoidal space, resulting in abnormal quantity and composition of extracellular matrix [1], which in turn leads to hepatitis, liver fibrosis, and cirrhosis. Thus, it is important to suppress hepatic inflammation in the early stages of liver fibrosis.

Oxidative stress, in particular, lipid peroxidation, has been considered one of the major causes of liver damage and has been reported to be associated with HSC activation [2]. Lipid peroxidation may provoke liver damage by compromising the integrity of membranes and by inducing covalent binding of reactive intermediates to important antioxidants such as glutathione [3]. Antioxidants are potent free radical scavengers and have been documented to protect hepatocytes from lipid peroxidation in the carbon tetrachloride- (CCl_4_-) or dimethylnitrosamine- (DMN-) induced liver injury models [4, 5]. Therefore, blocking or retarding the reactions of lipid peroxidation and the inflammatory process by antioxidants could be a promising therapeutic intervention for prevention or treatment of liver injuries.

Dietary phytochemicals of fruits, vegetables, whole grains, and other plant foods were shown to have potent antioxidant activity, and the mixture or combination of phytochemicals was proposed to be responsible for their strong antioxidant activity [6]. In most cases, the poor absorption rate of polyphenolic substances limits their use as dietary supplements in human. Particularly, many polyphenols exist in polymeric forms of high molecular weight that may further decrease their bioavailability [7]. Oligonol is a phenolic product derived from lychee fruit (*Litchi chinensis* Sonn.) extract by a manufacturing process that converts polyphenol polymers into oligomers. Oligonol comprises 16.0% monomers (catechin, epicatechin, epicatechin gallate, and epigallocatechin gallate), 13.9% dimers (procyanidin A1, A2, B1, and B2), and oligomers of proanthocyanidins [8]. Oligonol delivers higher levels of oligomeric proanthocyanidins compared with fruit and plant sources that contain high molecular weight proanthocyanidins [7].

There is a growing evidence that oligonol can elicit some physiological and biochemical alterations* in vitro* and* in vivo*, such as inhibition of adipogenesis in 3T3-L1 adipocytes [9], improvement of memory and cognition under an amyloid beta-induced Alzheimer's mouse model [10], the induction of apoptosis in MCF-7 and MDA-MB-231 human breast cancer cell lines [11], and antioxidant and anti-inflammatory effects in ultraviolet B- (UVB-) irradiated mouse skin [12]. However, the potential protective activity and mechanism of oligonol on acute liver inflammation induced CCl_4_ have not yet been conducted.

Here, we report that oral administration of oligonol exerts an antioxidant, anti-inflammatory, and hepatoprotective effect in CCl_4_-induced acute liver injury in rats. The possible molecular mechanism of action of oligonol was explored by analyzing the expression of nuclear factor-kappa B (NF-*κ*B), TNF-*α*, IL-1*β*, cyclooxygenase-2 (COX-2), inducible nitric oxide synthase (iNOS), Akt, and mitogen-activated protein kinases (MAPKs), including extracellular signal-regulated kinase (ERK), c-Jun NH_2_-terminal kinase (JNK), and p38.

## 2. Materials and Methods

### 2.1. Chemicals

Bicinchoninic acid (BCA) solution, bovine serum albumin (BSA),* t*-butyl hydroperoxide (*t*-BHP), butylated hydroxytoluene (BHT), CCl_4_, 1,1-diphenyl-2-picrylhydrazyl (DPPH), formalin, sodium carboxymethylcellulose (CMC), 2′,7′-dichlorofluorescin diacetate (DCFDA), phosphoric acid, trichloroacetic acid (TCA), 3-(4,5-dimethylthiazol-2-yl)-2,5-diphenyl-tetrazolium bromides (MTT), and Trolox were obtained from Sigma-Aldrich Co. 2-Thiobarbituric acid (TBA) was obtained from Tokyo Chemical Industry Co. Malondialdehyde (MDA) tetrabutylammonium salt was obtained from Fluka. Oligonol is commercially available (Amino Up Chemical Co., Ltd., Sapporo, Japan).

### 2.2. DPPH Assay

The scavenging activity of oligonol was measured using the stable DPPH free radical, according to a published method [13] with slight modifications. The reaction mixture contained 50 mM phosphate buffer at pH 7.4 (80 *μ*L), 100 *μ*M DPPH dissolved in ethanol (100 *μ*L), and the indicated concentrations of oligonol and Trolox (20 *μ*L). Triplicate reaction tubes were wrapped in aluminum foil and placed at room temperature for 30 min in the dark. Spectrophotometric readings were taken at 517 nm using a microplate reader (Apollo-LB913, Berthold Technologies). The percent inhibition of free radical production was calculated from ([*A*_0_ − (*A* − *A*_*b*_)]/*A*_0_) × 100, where *A*_0_ is the absorbance of the control, *A* is the absorbance of the sample, and *A*_*b*_ is the absorbance of the blank sample (containing all reagents except DPPH). IC_50_ values were obtained from the inhibition curves.

### 2.3. Antioxidative Activity against Lipid Peroxidation Induced FeSO_4_/H_2_O_2_ in Rat Liver Homogenates

Lipid peroxidation in rat liver homogenates induced by the Fenton reaction, comprising 0.1 mM FeSO_4_, 3 mM H_2_O_2_, various concentrations of the tested substances, and liver homogenates (7.5 mg protein/mL), was measured by the method of Buege and Aust [14] with some modifications. The reaction was started by the addition of FeSO_4_ and H_2_O_2_ and then incubated at 37°C for 10 min. The reaction was stopped by mixing with 3 mL of a stock solution of 15% (w/v) TCA, 0.375% (w/v) TBA, 0.125 M hydrochloric acid, and 0.6 mM BHT. The combination of reaction mixture and stock solution was heated for 30 min in a boiling water bath. After cooling, the flocculent precipitate was removed by centrifugation at 1,250 g for 20 min. The absorbance of the supernatant was determined at 532 nm, and the MDA concentration was calculated using MDA tetrabutylammonium salt as a standard. Protein concentrations were determined by the BCA assay using BSA as the reference standard.

### 2.4. Protective Effect of Oligonol on Cell Damage Induced by *t*-BHP

The human hepatocellular carcinoma cell line HepG2 was purchased from the Korean Cell Line Bank. The cells were cultured in a complete medium composed of Dulbecco's modified Eagle's medium (DMEM) supplemented with 10% fetal bovine serum (FBS), 1% glutamine, penicillin (100 *μ*g/mL), and streptomycin (100 *μ*g/mL) at 37°C in a 5% CO_2_ humidified incubated environment. The cells were placed in 96-well plates at a density of 2.0 × 10^4^ cells per well. After 24 h cultivation, the complete medium of the plates was replaced with serum-free medium, and various concentrations of oligonol (0.5, 2, 5, and 10 *μ*g/mL) were added to the cells. Four hours later, the cells were exposed to 300 *μ*M* t*-BHP for 3 h. After incubation, 100 *μ*L of MTT solution (1 mg/mL in phosphate buffered saline) was added to each well and incubated for another 2 h. After the culture medium had been removed, 100 *μ*L of DMSO was added and mixed to dissolve the MTT formazan crystals. The plates were read on a microplate reader (Apollo-LB913, Berthold Technologies) using a wavelength of 540 nm. The survival values, used to examine the protective effects of the compounds against cell damage by *t*-BHP, were expressed as a percentage of the absorbance of the normal cells.

### 2.5. Cell Lysis

Cells were washed by phosphate buffered saline (PBS), and then 1 mL of ice-cold PBS was added. Pellets were harvested at 1,000 g at 4°C for 3 min. The pellets were suspended in ProEX CETi Lysis Buffer (TransLab), incubated on ice for 20 min and then centrifuge at 14,000 g at 4°C for 10 min. The supernatants were used as total protein extraction.

### 2.6. Animals

Male Sprague-Dawley rats were obtained from Samtako (Osan, Korea). Animals were provided standard rat chow with free access to tap water and were maintained at a controlled temperature (23 ± 3°C) and humidity (50 ± 20%) with a 12 h light-dark cycle. With respect to ethical issues and scientific care, the animal protocol used in this study was reviewed and approved by the Pusan National University-Institutional Animal Care and Use Committee (PNU-IACUC; Approval number PNU 2008-0541).

### 2.7. Induction of Acute Hepatic Inflammation with CCl_4_

Twenty-four rats weighing 140–160 g and 5-6 weeks in age were assigned to 4 groups (*n* = 6): control, CCl_4_, Oli10, and Oli50. Animals in the control group received olive oil (CCl_4_ vehicle) by intraperitoneal (i.p.) injection and CMC (oligonol vehicle) by oral gavage; the CCl_4_ group received CCl_4_ and CMC, while the Oli10 and Oli50 group received CCl_4_ and oligonol at 10 and 50 mg/kg/day, respectively. Liver injury was induced by a single i.p. injection of 25% (w/v) CCl_4_ (0.6 g/kg body weight) in olive oil. Oligonol was suspended in 0.5% CMC solution to a concentration of 10 and 50 mg/mL and administered by oral gavage twice, once at 16 h and once at 30 min before CCl_4_ intoxication. Twenty-four hours after the CCl_4_ injection, all rats were euthanized by ether anesthesia, and the livers were excised and weighed. Blood samples for biochemical analyzes were obtained from the inferior vena cava.

### 2.8. Liver Homogenate Preparation

The remaining liver tissue was rapidly cut into small pieces and homogenized with two volumes (w/v) of ice-cold potassium phosphate buffer (pH 7.4) using an IKA T10 basic Ultra-Tur Rax homogenizer. Debris and nuclei were removed from the homogenate by centrifugation at 700 ×g at 4°C for 10 min and stored at −80°C for further analysis.

### 2.9. Histology

Liver specimens were fixed by immersion in 10% neutral buffered formaldehyde solution (NBF) for 24 h and then washed overnight. The samples from each group (*n* = 6) were dehydrated in a graded series of ethanol solutions, cleared in xylene, and embedded in paraffin. Eight to ten tissue sections (6 *μ*m thick) were cut and stained with hematoxylin and eosin (H&E) to assess the architectural alterations. The degree of liver damage was evaluated semiquantitatively using the Ishak system under a light microscope [15].

### 2.10. Biochemical Analysis of Liver Enzymes

Serum aspartate transaminase (AST) and alanine transaminase (ALT) activities were measured using the method described by Reitman and Frankel [16], using AST/ALT kits (Asan Chemical Co.).

### 2.11. Determination of MDA Content

MDA levels in the liver tissue were measured by a published method with modifications [17]. Standards were prepared via serial dilution of a stock solution of 10 *μ*M MDA tetrabutylammonium salt in distilled water. For the assay, 10 mg of liver tissue was homogenized with 1 mL of solution containing 26 mM TBA, 0.64 mM BHT, 0.93 M TCA, and 11 mM hydrochloric acid. The homogenates were heated for 1 h in a boiling water bath. After cooling, tubes were centrifuged for 15 min at 2,000 ×g. The absorbance of supernatant was determined at 532 nm and the MDA concentration was calculated using MDA tetrabutylammonium salt as a standard.

### 2.12. Measurement of ROS Level

A fluorometric assay was used to determine levels of ROS, such as ^•^O_2_^−^, ^•^OH, and H_2_O_2_. Nonfluorescent DCFDA was oxidized to the highly fluorescent 2′,7′-dichlorofluorescin (DCF) in the presence of esterases and ROS, including lipid peroxides [18]. For the assay, 50 *μ*M DCFDA was added to liver homogenates for 250 *μ*L of final volume. Changes in fluorescence intensity were measured every 5 min for 30 min on a fluorescence plate reader, GENios (Tecan Instrument, Salzburg, Austria), with excitation and emission wavelengths set at 485 and 530 nm, respectively.

### 2.13. RNA Extraction and Reverse Transcriptase-Polymerase Chain Reaction (RT-PCR)

Total RNA was extracted from samples of 100 mg of frozen liver by homogenization in Trizol reagent (Invitrogen). RNA purity was assessed by the absorbance ratio at 260 nm and 280 nm. cDNA was prepared from samples of 1 *μ*g of RNA with iScript cDNA Synthesis Kit (Bio-Rad, Hercules, CA, USA) according to the protocol provided by the manufacturer. PCR was performed in 20 *μ*L of reaction solution containing 1 *μ*g of cDNA and the appropriate primers from Bioneer (Daejeon, Korea) (Table 1) using a Promega GoTaq Flexi DNA Polymerase PCR kit. PCR conditions were as follows: denaturation at 95°C for 10 min, 35 cycles of 30 s at 95°C, 90 s at 60°C, 60 s at 72°C, and a final extension at 72°C for 5 min. GAPDH was measured as an internal control for normalization of mRNA levels. The amplified products were analyzed by 1.5% agarose gel electrophoresis and visualized by ethidium bromide staining under UV light illumination (Gel Doc/ChemiDoc imager, Azure). All reactions were performed in triplicate.

### 2.14. Western Blotting Analysis

Nuclear extracts, cytosol extracts, or total proteins of liver tissue were prepared as a published method with modification [19]. The protein concentration was measured by the BCA assay. Aliquots of protein (30 *μ*g) were denatured at 95°C for 5 min before electrophoresis on 10% SDS-polyacrylamide gel. After transfer to a polyvinylidene difluoride (PVDF) membrane (Millipore), the blot was blocked with 5% nonfat milk solution for 1 h at room temperature and then incubated with a 1 : 1,000 dilution of primary antibodies selective against either NF-*κ*B p65, total JNK, p-JNK, total ERK, p-ERK, total Akt, p-Akt, total p38, p-p38, histone, or *β*-actin (Santa Cruz Biotechnology) in Tris-buffered saline Tween-20 (TBST) at 4°C overnight, followed by 1 h at room temperature. The membrane was washed 3 times for 5 min each with TBST solution. The membranes were incubated with 1 : 10,000 dilution of horseradish peroxidase-conjugated rabbit or mouse secondary antibodies (Santa Cruz Biotechnology) at room temperature for 1 h. The transferred proteins were visualized with an enhanced chemiluminescence (ECL) detection system and the band intensities were determined using a Gel Doc/ChemiDoc imager (Azure). The protein concentration was determined with a BCA protein assay kit (Pierce, Rockford, IL, USA).

### 2.15. Statistical Analyses

All results are expressed as the mean ± SE of the indicated number of replicates. Data were analyzed for statistical differences by one-way analysis of variance (ANOVA). A *p value* of 0.05 or less was considered statistically significant.

## 3. Results

### 3.1. Antioxidative Activities of Oligonol against the Lipid Peroxidation of Rat Liver Homogenates Induced by FeSO_4_ and H_2_O_2_ and against DPPH Radical

The antioxidant activities of oligonol were investigated by the examination of the inhibitory effect against FeSO_4_/H_2_O_2_-induced lipid peroxidation in rat liver homogenates (Table 2) and the DPPH radical scavenging effect (Table 3). As positive control for the inhibition of lipid peroxidation, a well-known antioxidant BHT was tested. Under the reaction condition which allows the IC_50_ of BHT to be 15.01 *μ*M, IC_50_ of oligonol was 15.15 *μ*g/mL. Oligonol showed DPPH free radical scavenging activity (IC_50_ = 50.5 *μ*g) and Trolox was tested as a positive control (IC_50_ = 18.6 *μ*M)

### 3.2. Hepatoprotective Effect of Oligonol against Cell Damage Induced by *t*-BHP

**Figure 1:**
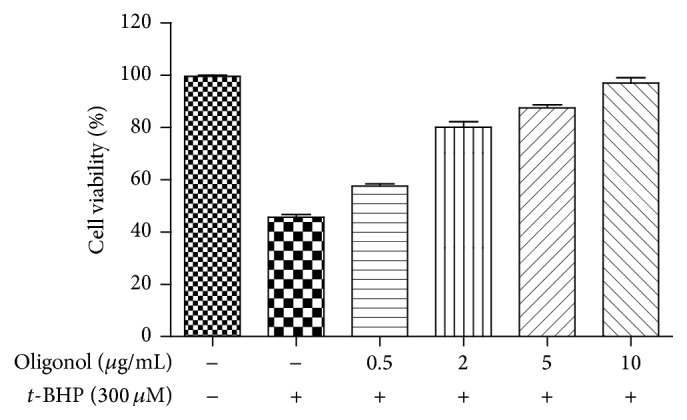
Effect of oligonol on HepG2 cell damage induced by *t*-BHP. Cell viability was assessed using MTT assays. Data shown represent means ± standard deviation of triplicate experiments.

**Figure 2:**
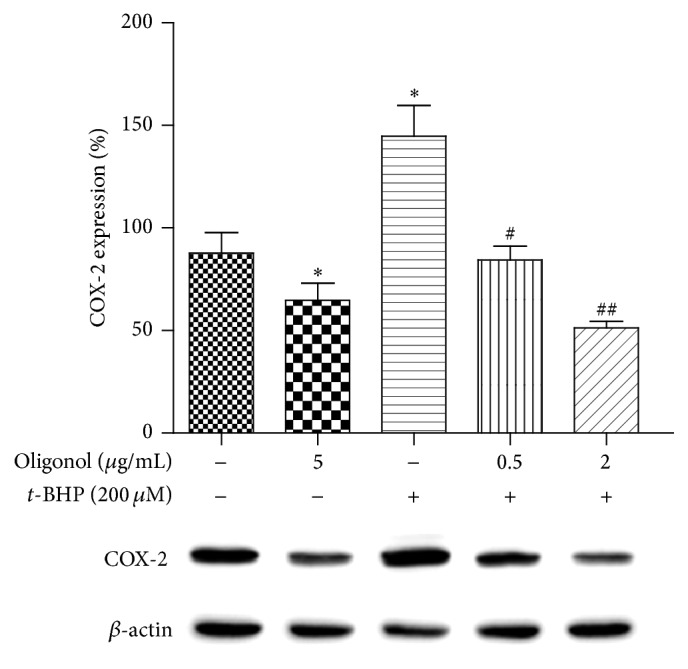
Effect of oligonol on *t*-BHP-induced COX-2 expression on HepG2 cells. HepG2 cells were treated with different concentrations of oligonol (0, 0.5, and 2 *μ*g/mL) for 4 h before being exposed to* t*-BHP (200 *μ*M) for 24 h. Western blotting was performed to detect COX-2 in whole protein (30 *μ*g) from HepG2 cell. One representative blot of each protein is shown from three experiments that yielded similar result, respectively. Values are normalized as percentage of *β*-actin. Values are mean ± SE of *n* = 3. ^*∗*^ *p* < 0.05 compared with the control group, and ^#^ *p* < 0.05 and ^##^ *p* < 0.01 compared with the* t*-BHP group.

*t*-BHP is a cytotoxic agent that is metabolized to free radicals including* t*-butoxyl,* t*-butylperoxyl, or methyl radical that interfere with cellular functions. The protective effect of oligonol on cell damage induced by *t*-BHP was examined (Figure 1). By exposing the cells to 300 *μ*M *t*-BHP for 3 h, cell viability decreased to 46%. However, oligonol was found to protect* t*-BHP-induced cell damage dose-dependently, and the EC_50_ was calculated to be 0.25 *μ*g. In addition, we investigated the effect of oligonol on the induction of proinflammatory mediator COX-2 in *t*-BHP-treated HepG2 cells at the protein level by western blot analysis. As shown in Figure 2, oligonol suppressed the *t*-BHP-induced COX-2 induction.

### 3.3. Changes in Body and Liver Weight and Serum Parameters in Rats Intoxicated by CCl_4_

**Figure 3:**
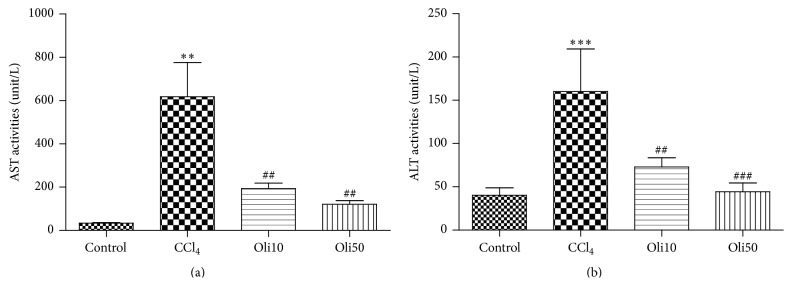
Measurements of serum ALT and serum AST levels. Groups are as described in “Methods.” Values are mean ± SE of *n* = 6 rats/group. ^*∗∗*^ *p* < 0.01 and ^*∗∗∗*^ *p* < 0.001 compared with the control group and ^##^ *p* < 0.01 and ^###^ *p* < 0.001 compared with the CCl_4_ group.

Treatment with CCl_4_ caused a slight increase in the ratio of body weight/liver weight when compared with the control group (Table 4). In contrast, animals injected with CCl_4_ and orally administered with oligonol showed significantly reduced ratios of liver weight to body weight, compared to untreated animals injected with CCl_4_. These results indicate that oligonol reduces the ratios of body to liver weight induced by CCl_4_ intoxication. Biochemical analyses of serum AST and serum ALT activities were performed to determine whether oligonol protected the liver from CCl_4_-induced injury (Figures 3(a) and 3(b)). Serum AST and serum ALT levels were significantly higher in rats injected with CCl_4_ (475.1 ± 330.3 and 160.0 ± 120.6 U/L, resp.) than in the control rats (35.5 ± 5.2 and 28.4 ± 4.3 U/L, resp.). Serum AST and serum ALT activities were significantly reduced by oral administration of oligonol at both 10 and 50 mg/kg doses; levels of AST and ALT in the Oli10 group were 193.7 ± 61.7 and 72.9 ± 26.0 U/L, respectively, and levels in the Oli50 group were 121.1 ± 29.4 and 44.2 ± 17.6 U/L, respectively.

### 3.4. Prevention of ROS Production and Lipid Peroxidation by Oligonol

**Figure 4:**
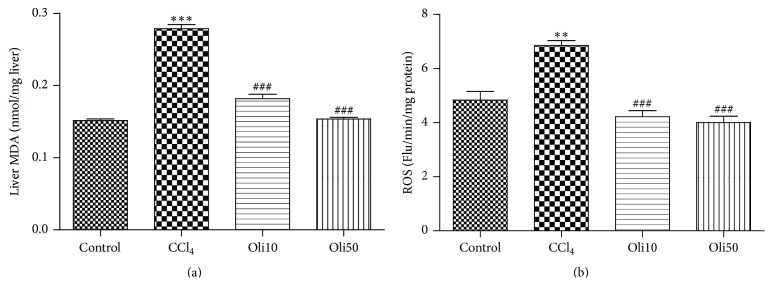
Effect of oligonol on ROS and MDA levels in rat liver intoxicated with CCl_4_. ROS generation was measured by DCF formation with a fluorescent probe, DCFDA. Effect of oligonol on CCl_4_-induced lipid peroxidation activity from the liver was measured by the method of Buege and Aust. Values are mean ± SE of *n* = 6 rats/group. ^*∗∗*^ *p* < 0.01 and ^*∗∗∗*^ *p* < 0.001 compared with the control group. ^###^ *p* < 0.001 compared with the CCl_4_ group.

To assess the overall oxidative status, total ROS was measured with DCFDA probe in the liver homogenates. Results show that increased ROS levels with CCl_4_ intoxication were suppressed by the administration of oligonol (Figure 4(a)). Induction of lipid peroxidation by CCl_4_ was measured by the production of MDA in liver tissues (Figure 4(b)). The MDA content in the livers of CCl_4_-treated rats was significantly higher than that in the control animals but significantly reduced in the livers of rats treated with oligonol in a dose-dependent manner, which is consistent with the results of ROS production and the liver function tests.

### 3.5. Liver Histopathology

**Figure 5:**
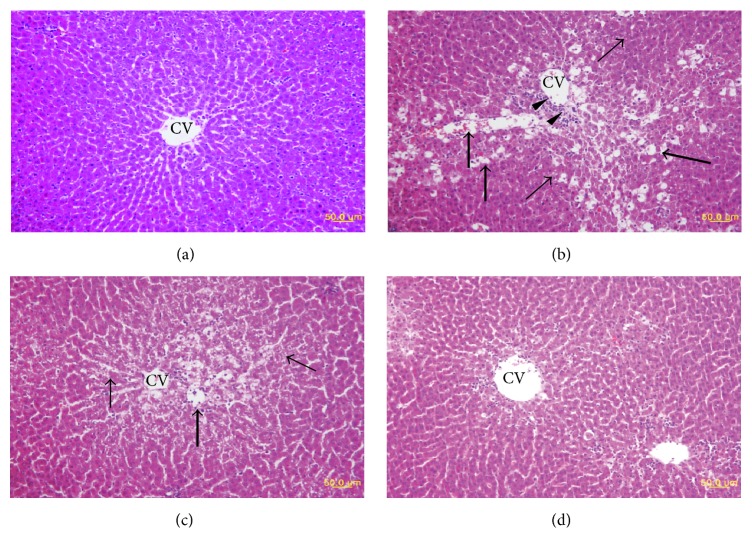
Effects of oligonol on CCl_4_-induced histopathological changes in rat livers. Representative H&E stained sections of livers of rats treated as described in methods. (a) Liver section of control group shows normal liver architecture, intact hepatocytes, and radiating hepatic cords from the central vein (CV); (b) CCl_4_-induced damage is indicated by distortion of the tissue architecture, submassive necrosis (thick arrows), fatty changes (thin arrows) in hepatocytes, and aggregations of inflammatory cells (arrowheads); (c) oligonol (10 mg/kg) plus CCl_4_; and (d) oligonol (50 mg/kg) plus CCl_4_; oligonol treatment reduced the pathological alterations induced by CCl_4_. All images are original magnification ×400.

The effect of oligonol on CCl_4_-induced histopathological changes in the liver was evaluated on H&E stained liver sections (Table 5). Livers of the control group showed normal lobular architecture with central veins and radiating hepatic cords. No histological abnormalities were observed (Figure 5(a)). In contrast, the liver sections from CCl_4_-treated animals showed distorted tissue architecture, submassive necrosis, vacuolization, and macrovesicular fatty changes of hepatocytes (Figure 5(b)). Notably, these pathologic changes were markedly reduced dose-dependently in the livers of animals treated with oligonol (Figures 5(c) and 5(d)).

### 3.6. Expression of TNF-*α*, IL-1*β*, COX-2, and iNOS mRNA

**Figure 6:**
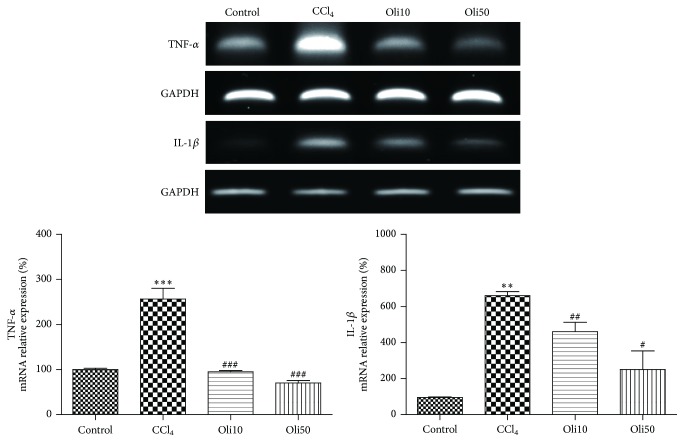
Effects of oligonol on TNF-*α* and IL-1*β* mRNA expression after CCl_4_ administration. RT-PCR was performed to measure TNF-*α* and IL-1*β* mRNA expression in the liver tissues. Values are mean ± SE of *n* = 6. ^*∗∗*^ *p* < 0.01 and ^*∗∗∗*^ *p* < 0.001 compared with the control group and ^##^ *p* < 0.01 and ^###^ *p* < 0.001 compared with the CCl_4_ group.

**Figure 7:**
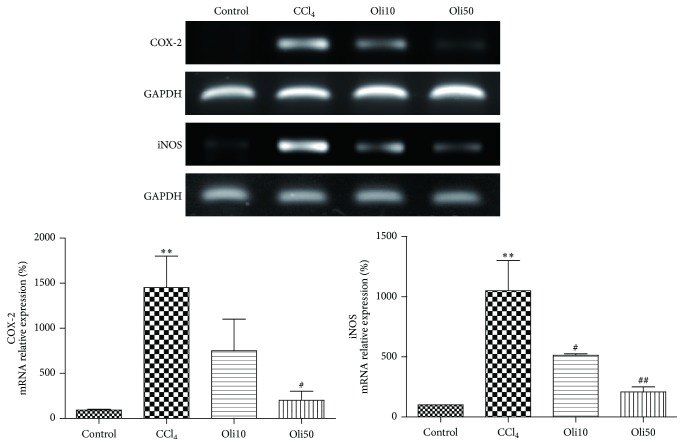
Effects of oligonol on COX-2 and iNOS mRNA expression after CCl_4_ administration. RT-PCR was performed to measure COX-2 and iNOS mRNA expression in the liver tissues. Values are mean ± SE of *n* = 6. ^*∗∗*^ *p* < 0.01 compared with the control group and ^#^ *p* < 0.05 and ^##^ *p* < 0.01 compared with the CCl_4_ group.

Expression of mRNA of proinflammatory cytokines TNF-*α* and IL-1*β* and proinflammatory proteins COX-2 and iNOS in the liver was measured by RT-PCR. Following agarose gel electrophoresis of reaction products, mRNA levels were quantified by normalization against the expression of the housekeeping gene, GAPDH. CCl_4_ treatment increased the expression of TNF-*α*, IL-1*β*, COX-2, and iNOS mRNA in the liver, but these were dose-dependently and significantly reduced by pretreatment of rats with oligonol (Figures 6 and 7).

### 3.7. NF-*κ*B Translocation to the Nucleus

**Figure 8:**
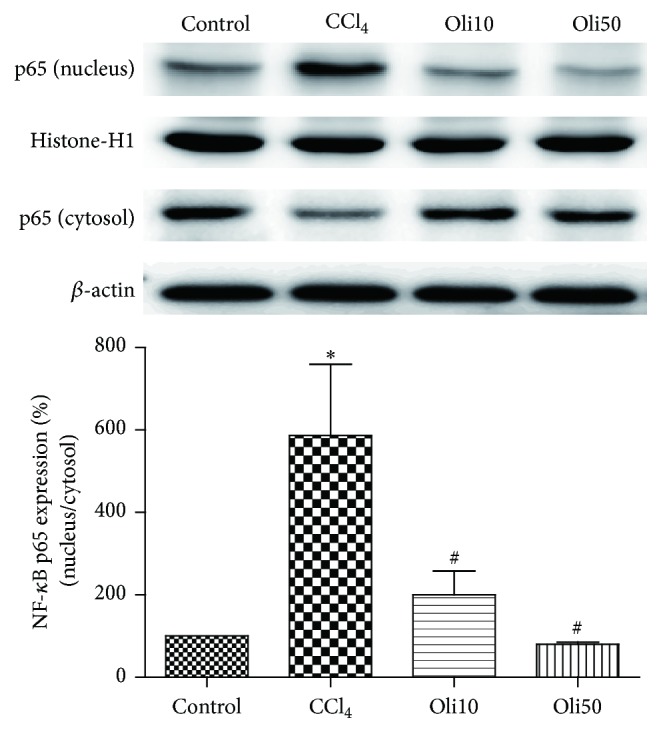
Effects of oligonol on CCl_4_-induced NF-*κ*B p65 activation. Western blotting was performed to detect nuclear and cytoplasmic localization of NF-*κ*B p65 in the livers tissues. The relative level of NF-*κ*B p65 in the nuclear and cytosol compared with *β*-actin and histon, respectively, and quantified by image analysis. Values are mean ± SE of *n* = 6. ^*∗*^ *p* < 0.05 compared with the control group and ^#^ *p* < 0.05 compared with the CCl_4_ group.

The protein level of transcription factor NF-*κ*B was examined. Activation of NF-*κ*B was based on the detection of its translocation into cell nuclei from its initial location in the cytoplasm where it exists in an inactive form. Western blotting of NF-*κ*B p65 protein in nuclear and cytosolic fractions of the liver tissues indicates that CCl_4_ treatment exhibited an enhancement of nuclear NF-*κ*B and a reduction of cytosolic NF-*κ*B (Figure 8). Oligonol treatment to CCl_4_-intoxicated rat markedly inhibited CCl_4_-induced increase of NF-*κ*B in the nuclear fraction of the liver. It also abrogated the reduction of NF-*κ*B in the cytosolic fraction. The relative level of NF-*κ*B p65 in the nuclear and cytosol is compared with *β*-actin and histon, respectively, and quantified by image analysis. It showed that CCl_4_-treated rats had significantly increased expression of NF-*κ*B p65 compared with the control animals. Moreover, the expression of NF-*κ*B p65 in the oligonol-treated rats was reduced significantly in a dose-dependent manner. These results demonstrate that oligonol treatment in CCl_4_-intoxicated rats strongly inhibited the translocation of NF-*κ*B p65 from the cytosol to the nuclear fraction.

### 3.8. MAPKs and Akt Signaling Pathways Involved in NF-*κ*B Activation

**Figure 9:**
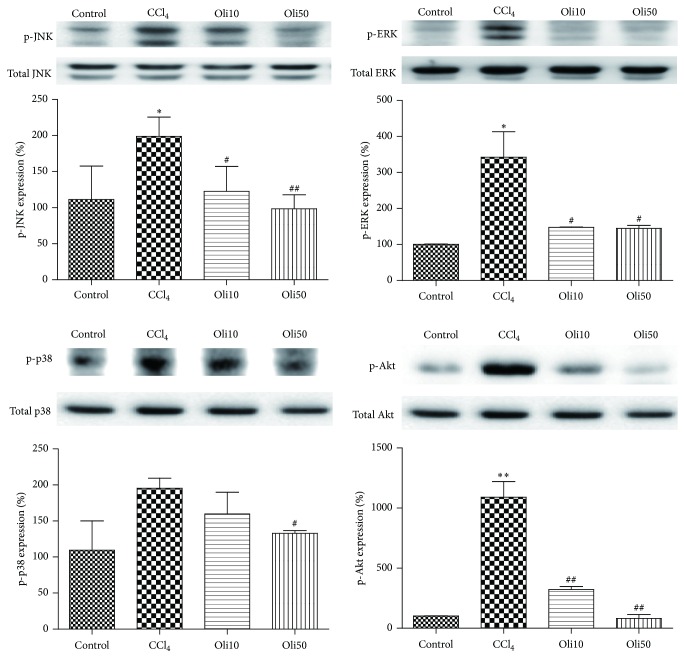
Effects of oligonol on CCl_4_-induced MAPKs and Akt activation. Western blotting was performed to detect ERK, JNK, and p38 MAPK, as well as Akt, in liver tissues. Activation of these kinases was detected using their specific phosphorylated antibody. The band intensity of phosphorylated forms of MAPKs and Akt was quantified by densitometry and normalized to total forms of MAPKs and Akt, respectively. Values are mean ± SE of *n* = 6. ^*∗*^ *p* < 0.05 and ^*∗∗*^ *p* < 0.01 compared with the control group and ^#^ *p* < 0.05 and ^##^ *p* < 0.01 compared with the CCl_4_ group.

To investigate the molecular mechanism of NF-*κ*B activation in the CCl_4_-intoxicated rat, we measured the expression levels of ERK1/2, JNK, and p38 MAPKs as well as Akt by using western blot analysis. The phosphorylation of MAPKs and Akt was increased in rats treated with CCl_4_ alone as compared with the control group. However, treatment with low (10 mg/kg/day) and high (50 mg/kg/day) dose of oligonol in CCl_4_-intoxicated rats significantly decreased the expression levels of phosphorylated ERK1/2, JNK, and p38 MAPKs as well as Akt, in a dose-dependent manner (Figure 9).

## 4. Discussion

Oxidative stress, caused by the increased production of reactive oxygen species (ROS), is thought to be a key risk factor in the development of liver disease [20]. CCl_4_ intoxication has been widely used as an experimental model of liver injury. CCl_4_ is a substrate for cytochrome P450 2E1 (CYP2E1). It is converted to a CCl_3_ radical, which generates CCl_3_OO^•^ by reacting with molecular oxygen. Since CCl_3_OO^•^ reacts with microsomal membranes and induces lipid peroxidation, membrane damage by free radical chain reaction has been postulated to be the primary cause of hepatocellular injury by this compound [21]. In CCl_4_-induced injury, antioxidants are widely known to be able to protect against hepatocyte necrosis because they intercept the CCl_4_-induced oxidative stress in hepatocytes by scavenging ^•^CCl_3_ and lipid peroxy radicals [22].

In this study, a single dose of CCl_4_ induced distorted tissue architecture, submassive centrizonal necrosis, fatty changes, and inflammatory cell infiltration. However, these pathologic changes were significantly reduced by pretreatment of 10 and 50 mg/kg of oligonol dose-dependently. Treatment of oligonol effectively improved the CCl_4_-induced elevation in serum AST and serum ALT levels, indicating the hepatoprotective effects of oligonol against CCl_4_ intoxication. CCl_4_ treatment caused high levels of liver oxidative damage, as evidenced by a significant elevation of ROS production and MDA concentration in liver homogenates. However, oligonol markedly inhibits CCl_4_-induced oxidative stress in liver of rats in a dose-dependent manner.

Antioxidant activity of oligonol is well established in many studies [23], and the activities were consistent with the results that oligonol exerts the inhibitory effect against FeSO_4_/H_2_O_2_-induced lipid peroxidation in rat liver homogenates and against the DPPH radical scavenging effects. In addition, the protective effect of oligonol via antioxidative activity was assessed in *t*-BHP-induced HepG2 cell damage. HepG2 cells are considered a reasonable model for studying* in vitro* xenobiotic metabolism and liver toxicity since they maintain a majority of specialized functions similar to normal human hepatocytes [24]. The oxidant *t*-BHP is well known to induce oxidative stress [25]. Oligonol showed hepatoprotective effects against *t*-BHP-induced oxidative stress. Taken together, hepatoprotective effects of oligonol in CCl_4_-intoxicated rat model and in *t*-BHP-induced HepG2 cells may be due to the potent antioxidant and free radical scavenging activities of oligonol.

Proinflammatory cytokines such as TNF-*α*, IL-1*β*, and IL-6 have been the focus of investigations of inflammatory organ injury because the uncontrolled and prolonged action of these proteins is potentially harmful [26]. Considerable evidence suggests that TNF-*α* and IL-1*β* contribute to the pathogenesis of liver inflammatory diseases by activating the NF-*κ*B signaling pathway [27], suggesting that it may be important to monitor proinflammatory cytokines when studying liver injury. In our study, we focused on the anti-inflammatory effect of oligonol by analyzing the expression of NF-*κ*B p65, TNF-*α*, and IL-1*β*. Our data show that the production of TNF-*α* and IL-1*β* were significantly increased by CCl_4_-induced hepatotoxicity, which is consistent with the findings of Reyes-Gordillo et al. [28]. We found that the translocation of NF-*κ*B p65 protein into nucleus and TNF-*α* and IL-1*β* mRNA expression were inhibited in rat pretreated oligonol, suggesting that oligonol acts, at least in part, by inhibition of NF-*κ*B activity.

Beside proinflammatory cytokines, NF-*κ*B regulates the expression of the inflammatory proteins COX-2 and iNOS. This study showed a significant increase of COX-2 and iNOS mRNA expression levels in the liver treated CCl_4_. These increases were attenuated by treatment with oligonol. In the study with HepG2 cells, the increased COX-2 protein levels in *t*-BHP-treated cells were also reduced by pretreatment of oligonol in a dose-dependent manner. These results suggest that oligonol exerts effects in suppressing inflammatory responses caused by CCl_4_ or *t*-BHP.

A series of upstream kinases including MAPKs and Akt are involved in a relay in transmitting stimuli-induced signals to the downstream transcription factors like NF-*κ*B by regulating transcriptional activation of a variety of genes encoding COX-2, iNOS, TNF-*α*, and IL-1*β*. MAPKs mediate intracellular signaling associated with a variety of cellular activities including cell proliferation, differentiation, survival, death, and transformation [29]. The mammalian MAPK family consists of ERK, JNK, and p38. The MAPK pathways are activated by diverse extracellular and intracellular stimuli including cytokines and various cellular stressors such as oxidative stress caused ROS and endoplasmic reticulum stress. Activated MAPKs phosphorylate various substrate proteins including transcription factors like NF-*κ*B. Our results also showed that CCl_4_ exposure induced activation of MAPKs in rat liver, and oligonol inhibited CCl_4_-induced JNK, ERK, and p38 phosphorylation. In addition, in order to activate NF-*κ*B in response to specific stimuli, NF-*κ*B needs to first be liberated from its inhibitory I*κ*B partner [30]. On phosphorylation by I*κ*B kinase (IKK), I*κ*B is degraded by the proteasome and NF-*κ*B is set free. IKK can be activated by Akt. In this study, phosphorylation of Akt was increased in CCl_4_-intoxicated rats, while oligonol suppressed this Akt activation. This suggests that oligonol is involved with the Akt/NF-*κ*B pathway.

## 5. Conclusion

The findings of the present study indicate that oligonol is highly effective in preventing CCl_4_-induced acute liver damage, which is most likely mediated by its activity to suppress oxidative stress and lipid peroxidation as an antioxidant. It has capacity to inhibit NF-*κ*B p65 activation and the expression of the proinflammatory cytokines, TNF-*α* and IL-1*β*, and proinflammatory proteins, such as COX-2 and iNOS. The underlying mechanisms for this NF-*κ*B inactivation may be due to inhibition of the activation of upstream kinases including ERK, JNK, and p38 MAPKs as well as Akt. Our data suggests that oligonol may be useful as a therapeutic agent for the suppression of hepatic inflammation.

## Figures and Tables

**Table 1:** Oligonucleotide sequences used in RT-PCR analysis.

Group	Direction	Sequence
TNF-*α*	Forward	TTCTGTCTACTGAACTTGGGGGTGATCGGTCC
	Reverse	GTATGAGATAGCAAATCGGCTGACGGTGTGGG

IL-1*β*	Forward	ATGGCAACTGTTCCTGAACTCAACT
	Reverse	CAGGACAGGTATAGATTCTTTCCTTT

COX-2	Forward	CCAGAGCAGAGAGATGAAATACCA
	Reverse	GCAGGGCGGGATACAGTTC

iNOS	Forward	GATTCAGTGGTCCAACCTGCA
	Reverse	CGACCTGATGTTGCCACTGTT

GAPDH	Forward	GACAACTTTGGCATCGTGGA
	Reverse	ATGCAGGGATGATGTTCTGG

TNF-*α*: tumor necrosis factor-alpha; IL-1*β*: interleukin-1 beta; COX-2: cyclooxygenase-2; iNOS: inducible nitric oxide synthase; GAPDH: glyceraldehyde-3-phosphate dehydrogenase.

**Table 2:** Antioxidative activities of oligonol against DPPH radical.

Substances	Concentration	Inhibition (%)	IC_50_
Oligonol (*µ*g/mL)	5	10.7 ± 0.002	50.5
	10	28.5 ± 0.001	
	25	38.9 ± 0.001	
	50	49.9 ± 0.001	
	100	61.1 ± 0.006	

Trolox (*µ*M)	5	19.2 ± 0.008	18.6
	10	26.1 ± 0.003	
	25	50.4 ± 0.007	
	50	65.1 ± 0.001	
	100	71.3 ± 0.006	

The reaction mixture consisted of 0.5 mL of 60 *µ*M ethanolic solution of DPPH and 0.5 mL of various concentrations of sample solution. After allowing the mixture to stand at room temperature for 30 min, the absorbance of the remaining DPPH was determined at 517 nm. Trolox was used as a positive control.

**Table 3:** Antioxidative activities of oligonol against the lipid peroxidation of rat liver homogenates induced by FeSO_4_ and H_2_O_2_.

Substances	Concentration	Inhibition (%)	IC_50_
Oligonol (*µ*g/mL)	10	47.52 ± 0.06	15.16
	50	62.01 ± 0.01	
	100	74.74 ± 0.06	
	500	93.02 ± 0.01	

BHT (*µ*M)	5	16.51 ± 0.04	15.01
	10	43.43 ± 0.03	
	20	59.82 ± 0.01	
	50	89.61 ± 0.05	

Results are expressed as mean ± SE (*n* = 3). The reaction mixture was composed of the rat liver homogenate, 0.1 mM FeSO_4_, 3 mM H_2_O_2_, and various concentrations of oligonol or BHT. After incubation at 37°C for 10 min, the amount of MDA formation was measured by the method of Buege and Aust. Inhibition (%) of MDA formation in oligonol or BHT was calculated based on the amount of MDA formation of the FeSO_4_/H_2_O_2_-treated control after subtracting the normal.

**Table 4:** Effects of oligonol on body and liver weights of rats treated with CCl_4_.

	Body weight (g)	Liver weight (g)	Ratio (%)^a^
Control	154.02 ± 7.40	5.96 ± 1.25	3.85
CCl_4_	158.01 ± 7.29	7.69 ± 0.66^*∗∗*^	4.87
Oli10	158.16 ± 4.62	6.98 ± 0.34^#^	4.42
Oli50	156.75 ± 10.05	6.71 ± 0.29^#^	4.29

CCl_4_: CCl_4_ alone treated group; Oli10: oligonol (10 mg/kg) with CCl_4_; Oli50: oligonol (50 mg/kg) with CCl_4_. ^a^Values are expressed as the ratios of liver weight to body weight. Data are the mean ± SE of *n* = 6 rats/group. ^*∗∗*^ *p* < 0.01 compared with the control group and ^#^ *p* < 0.05 compared with the CCl_4_ group.

**Table 5:** Effects of oligonol on the histopathological score of liver of CCl_4_-treated rats.

Group	Number	Histopathological score of liver				
		0	1	2	3	4
Control	6	6	0	0	0	0
CCl_4_	6	0	0	0	1	5
Oli10	6	1	2	1	2	0
Oli50	6	3	1	1	1	0
